# Spontaneous recovery of cochlear fibrocytes after severe degeneration caused by acute energy failure

**DOI:** 10.3389/fphar.2014.00198

**Published:** 2014-08-26

**Authors:** Kunio Mizutari

**Affiliations:** Department of Otolaryngology, National Defense Medical College, SaitamaJapan

**Keywords:** regeneration, cochlear lateral wall, fibrocyte, 3-nitropropionic acid (3-NP), endocochlear potential, delayed hearing recovery, acute energy failure

## Abstract

Cochlear fibrocytes in the lateral wall region play a critical role in the regulation of inner ear ion and fluid homeostasis, although these are non-sensory cells. Along with other non-sensory cells, fibrocytes in the spiral ligament have been reported to repopulate themselves after damage. However, the studies of regeneration of cochlear fibrocytes have been difficult because a suitable fibrocyte-specific degeneration model did not exist. Therefore, we analyzed cochlear fibrocytes using a rat model of acute cochlear energy failure induced by a mitochondrial toxin. This model is unique because hearing loss is caused by apoptosis of fibrocytes in the cochlear lateral wall not by damage to sensory cells. Although this model involves severe damage to the cochlear lateral wall, delayed spontaneous regeneration occurs without any treatment. Moreover, partial hearing recovery is accompanied by morphological remodeling of the cochlear lateral wall. Two hypotheses are conceivable regarding this spontaneous recovery of cochlear fibrocytes. One is that residual cochlear fibrocytes proliferate spontaneously, followed by remodeling of the functional region of the lateral wall. Another is that some foreign cells such as bone marrow-derived cells promote morphological and functional recovery of the lateral wall. Acceleration of the lateral wall recovery promoted by these mechanisms may be a new therapeutic strategy against hearing loss.

## INTRODUCTION

Hearing loss is one of the most common disabilities in the world, particularly in aged populations, and its prevalence is increasing. In the United States, 35% of individuals between 65 and 79 years old report hearing impairment; among those aged ≥80, the corresponding figure is 53% (Caban et al., 2005). Moreover, acute sensorineural hearing loss, such as sudden deafness, is a huge social problem because these diseases may occur at a younger age without any warning signs. Several etiological theories have been proposed regarding sudden deafness, such as cochlear ischemia (Seidman et al., 1999) or viral infection (Tucci, 2000). However, the etiology of these diseases remains unknown. In general, sensorineural hearing loss is irreversible once it has occurred because the loss of sensory hair cells and neurons is permanent in the mature mammalian cochlea (Kwan et al., 2009).

In addition to sensory cells, such as hair cells or spiral ganglion cells, non-sensory cells are very important to receive sound signals. For example, marginal, intermediate, and basal cells in the stria vascularis are important to maintain cochlear ion environment. Cochlear fibrocytes also play a critical role in the regulation of the inner ear ion and fluid homeostasis (Minowa et al., 1999; Delprat et al., 2005), although these are non-sensory cells. Cochlear fibrocytes of the spiral ligament contain Na^+^/K^+^-ATPase and Na^+^/K^+^/Cl^-^ cotransporters. These molecules are essential for ionic homeostasis and maintenance of the endocochlear potential (Schulte and Adams, 1989; Spicer and Schulte, 1996; Crouch et al., 1997; Adachi et al., 2013). Gap junctions also express between and among cochlear cells including fibrocytes. Gap junctions make recycling endolymphatic potassium ions pass through these cells, therefore gap junctions are also critical for maintenance of the endocochlear potential (Kikuchi et al., 1995). Apoptosis in the cochlear lateral wall is observed in an experimental model that involves aminoglycoside-induced hearing loss (Labbe et al., 2005) and in a presbyacusis model (Alam et al., 2001), where the hearing loss is mainly caused by damage to sensory cells. Degeneration of the lateral wall fibrocytes leads to hearing loss because of a decrease in the endocochlear potential (Gratton et al., 1996, 1997; Schmiedt et al., 2002).

It was also reported that fibrocytes in the spiral ligament are capable of repopulation after damage by noise or a drug (Roberson and Rubel, 1994; Yamashita et al., 1999; Hirose and Liberman, 2003; Lang et al., 2003). Under normal conditions, cochlear fibrocytes can continue to divide even when the animal is at an advanced age (Lang et al., 2003). On the other hand, the contribution of cochlear fibrocyte repopulation to hearing recovery is still unknown because there are no suitable experimental models that can help to evaluate the influence of the cochlear lateral wall on hearing loss.

## ACUTE ENERGY FAILURE IN THE INNER EAR AS A RESULT OF MITOCHONDRIAL INHIBITION

Hoya et al. (2004) reported a unique model of hearing loss in rats that involves mitochondrial inhibition in the inner ear. They used 3-nitropropionic acid (3-NP), which is an irreversible inhibitor of succinate dehydrogenase, a complex II enzyme of the mitochondrial electron transport chain (Alston et al., 1977; Coles et al., 1979). 3-NP was administered into the round window niche to inhibit ATP synthesis in the inner ear. This method of ATP deprivation in the inner ear is considered to replicate inner ear ischemia. Among several proposed etiologies of sudden deafness, inner ear ischemia is an important theory of the cause of this disease. In the detailed morphological and physiological studies of this model of hearing loss (Hoya et al., 2004; Okamoto et al., 2005; Mizutari et al., 2008), the main cause of hearing loss appears to be the loss of fibrocytes in the cochlear lateral wall, particularly in the spiral ligament. Degeneration of cochlear lateral wall fibrocytes is induced by apoptosis, and cochlear sensory cells, such as hair cells or spiral ganglion cells, survived even after severe hearing loss occurred (Mizutari et al., 2008). Another research group reported that 3-NP-induced hearing loss primarily occurs via a reduction of the endocochlear potential, along with a significant loss of spiral ligament fibrocytes (Kada et al., 2009).

This approach can cause severe and selective damage to fibrocytes in the cochlea without severe degeneration of sensory cells (Okamoto et al., 2005). Therefore, this animal model is considered to be an ideal platform for exploration of the morphological and functional prognosis of damaged cochlear fibrocytes.

## SPONTANEOUS RECOVERY OF FIBROCYTES IN THE COCHLEAR LATERAL WALL AFTER SEVERE DAMAGE

A recent study revealed that cochlear fibrocytes in the lateral wall can repopulate themselves after severe 3-NP-induced degenerative changes accompanied with profound hearing loss (Mizutari et al., 2011). This cochlear lateral wall remodeling is observed in the late phase, accompanied by partial hearing recovery. In general, sensorineural hearing loss, as a result of cochlear damage, is permanent after the acute phase. Therefore, studies of hearing protection from cochlear damage usually have been performed in the early phase or before cochlear damage. However, Mizutari et al. (2011) showed that delayed hearing recovery starts 2 months after the onset of hearing loss. Moreover, an increase of Na^+^/K^+^/ATPase-β1 expression and cell proliferation was proven by an experiment with 5-bromo-2^′^-deoxyuridine (BrdU). This type of delayed recovery of hearing is occasionally observed during treatment of sudden deafness in the clinic (Yeo et al., 2007).

Hearing recovery in the late phase in patients with sudden deafness is rarely detected, even if it occurs. The mechanism of clinical recovery of hearing remains unknown, but clinical course of the delayed recovery of hearing is similar to that involved in 3-NP-induced hearing loss. At least the spontaneous regeneration of hair cells or spiral ganglion cells never occurs because a loss of sensory cells is irreversible in mammals once these cells are gone (Kwan et al., 2009). Therefore, regeneration of non-sensory cells should play an important role in the reversal of this pathophysiology. Reduction of the endocochlear potential is promoted by disruption of cochlear blood flow, and this process is believed to be a cause of acute hearing loss (Shi, 2011). It is possible that a certain percentage of sudden deafness cases with delayed recovery of hearing is caused by an endocochlear potential reduction driven by fibrocyte dysfunction.

## THE MECHANISM OF FUNCTIONAL RECOVERY OF LATERAL WALL FIBROCYTES

Several mechanisms of lateral wall regeneration have been proposed. One is local proliferation of the fibrocytes that can survive the damage. It is reported that cochlear fibrocytes can continue to divide, although the proliferation capacity is reduced when the animal is at an advanced age (Lang et al., 2003). Moreover, Lang et al. (2003) reported that the proliferation capacity is increased after the cochlear lateral wall is damaged by furosemide. It is possible that is the main mechanism of the lateral-wall remodeling after damage. However, the self-renewal ability of cochlear fibrocytes appears to be unreliable for remodeling of all structures of the lateral wall when the loss of fibrocytes is severe.

An additional hypothesis is that some substance that migrates into the cochlear lateral wall accelerates the regeneration of fibrocytes. Several studies demonstrated that the spiral ligament contains bone marrow-derived cells that can differentiate into macrophages in a deafened cochlea after acoustic trauma (Hirose et al., 2005; Tan et al., 2008). Other reports showed that macrophages migrating into an injured site perform an important function in promoting regeneration after injury in various tissues such as retinal ganglion cells (Lorber et al., 2005), dorsal root ganglion cells, and cortical neurons (Gensel et al., 2009). A transplant of mesenchymal stem cells (MSCs) into a 3-NP-damaged cochlea promotes hearing recovery (Kamiya et al., 2007). In addition, the latter report showed that the main mechanism of fibrocyte repair is acceleration of fibrocyte regeneration driven by transplanted MSCs (Kamiya et al., 2007).

Therefore, acceleration of fibrocyte repopulation is believed to be a promising therapeutic strategy for treating some types of sensorineural hearing loss. Further research is expected to reveal the detailed mechanism behind the hearing recovery via fibrocyte regeneration.

## Conflict of Interest Statement

The author declares that the research was conducted in the absence of any commercial or financial relationships that could be construed as a potential conflict of interest.
